# Recent Advances and Future Perspectives in Additives and Technologies for Low-Density Drilling Fluids

**DOI:** 10.1021/acsomega.6c04580

**Published:** 2026-08-20

**Authors:** Raphael Dannemann-Pires, Claudio Roberto Duarte, Marina Seixas Pereira

**Affiliations:** 28119 Universidade Federal de Uberlândia, Chemical Engineering School, Uberlândia, State of Minas Gerais 38400-694, Brazil

## Abstract

This review investigates the evolution of additives and technologies developed for density reduction in drilling fluids and discusses their applicability and main trade-offs. The authors present the main advantages, limitations, and economic and environmental implications of each additive class and discuss their influence on fluid stability, rheology, and operational safety. A literature search was carried out in the ScienceDirect, Web of Science, and Scopus databases, using the following keywords: “low-density drilling fluid additives,” “lightweight drilling fluid additives,” “density reduction drilling fluid additives,” and “underbalanced drilling fluid additives,” covering experimental and review papers published between 2015 and 2026. A second search was performed using the same keywords, substituting “drilling fluid” with “drilling mud” to broaden the scope of the search. Most of the studies were experimental and focused on water-based systems, identifying hollow glass spheres as the most investigated and widely adopted additive for density reduction. Emerging materials such as polymeric and biobased additives also show promising potential for sustainable and low-cost formulations. Oil-based systems remain underrepresented in the literature despite the lower density of oil as a base fluid compared to water and the rise of environmentally friendly base oils. By clarifying technological trade-offs, identifying knowledge gaps, and outlining concrete innovation pathways, this review consolidates fragmented literature into a unified review specifically focused on density-reducing additives. In addition, by evaluating the technical, economic, and environmental factors involved, it supports the advancement of next-generation lightweight drilling fluids. Overall, the findings indicate that research on density-reducing additives is still limited, revealing a clear need for further studies and field-scale validation to support the development of the next generation of ultralightweight and sustainable drilling fluids.

## Graphical abstract

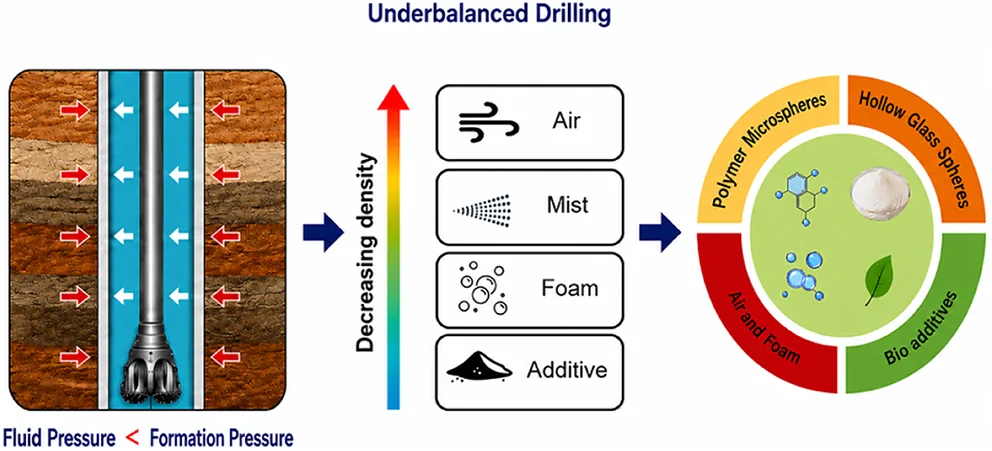

## 1 Introduction

Oil is a critical energy resource that fuels the global economy. It drives ground, maritime, and air transportation and serves as a fundamental feedstock for the production of plastics, fertilizers, pharmaceuticals, and numerous other essential goods. Moreover, oil holds significant geopolitical relevance, influencing international relations and the economic development of both producing and consuming nations.^1–3^ These factors reinforce the enduring importance of oil as both an economic driver and a geopolitical instrument in the global system.

Global forecasts indicate that oil demand will continue to expand in the coming decades, reinforcing its status as an indispensable energy source. According to the most recent data from the Organization of the Petroleum Exporting Countries (OPEC), global demand is projected to reach 112.3 million barrels per day (mb/d) by 2029, which means an increase of 10.1 mb/d relative to 2023. In the long term, demand is expected to rise from 102.2 mb/d in 2023 to 120.1 mb/d by 2050.^4^ These projections reflect the sector’s global significance and indicate the need for continuous research, strategic investment, and technological innovation to sustain growth and ensure future energy security.

Drilling, which involves the construction of wellbores to access subsurface petroleum reservoirs, is considered to be one of the most technologically advanced and high-risk stages in exploration projects. It has been estimated that drilling operations may account for more than half of the total cost of exploration and development activities, thereby playing a critical role in determining overall project feasibility.^5^ The technical challenges encountered, such as high pressures, unstable formations, and equipment failures, further contribute to escalating costs.^6–8^ In this context, drilling fluids play a crucial role in mitigating operational challenges and ensuring the efficiency and safety of the drilling process.^9^

Drilling fluids, also known as drilling muds, are complex mixtures injected into the wellbore to perform several critical functions during drilling operations, including the removal of drill cuttings, maintenance of hydrostatic pressure to prevent formation influxes, stabilization of wellbore walls, and cooling and lubrication of both the drill string and the bit.^10–12^ The efficiency of these functions is directly governed by the fluid composition and physicochemical properties, suggesting the need for continued research and development to design tailored formulations for specific drilling environments. The main functions of drilling fluids and their operating mechanisms are illustrated in Figure 1.

**Figure 1:**
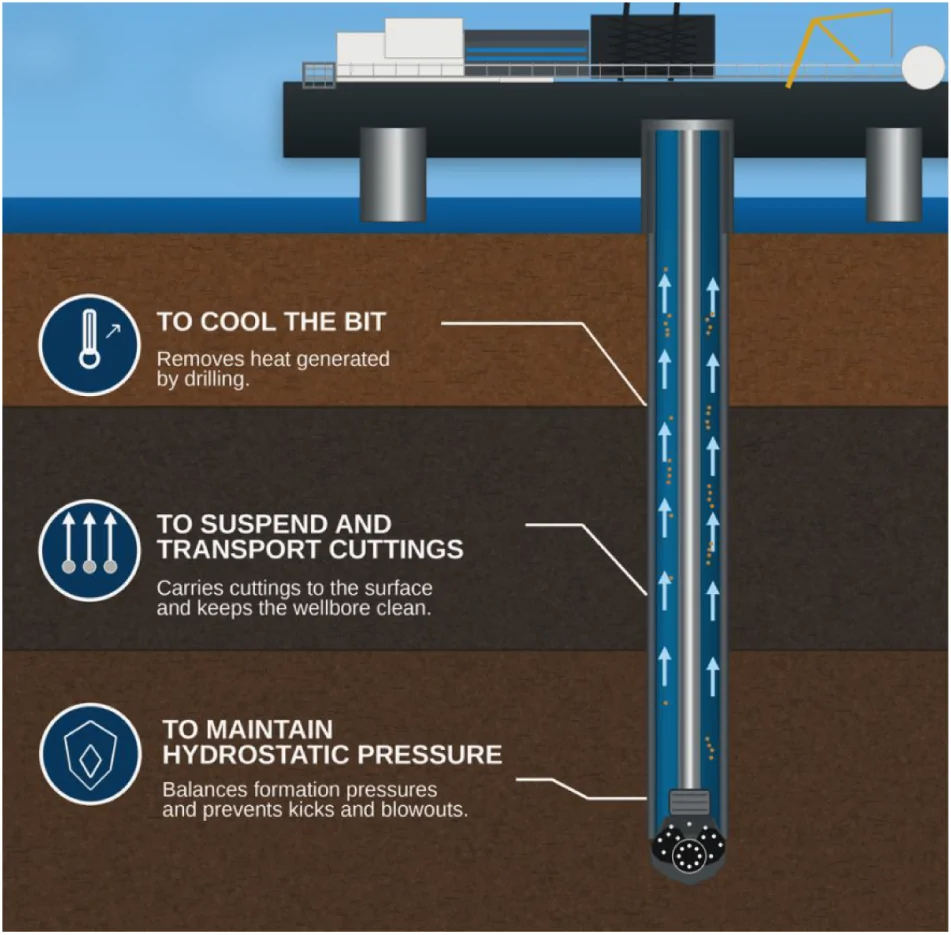
Main functions of drilling fluids.

Despite their frequent association with oil and gas extraction, drilling fluids are used in a broad range of drilling activities, including geothermal drilling, mineral exploration, water-well construction, and geotechnical investigations. Although these applications differ in their objectives and geological conditions, they share several technical challenges, such as fluid loss, wellbore instability, hole cleaning, and thermal degradation. Consequently, advances in drilling fluid technology can often be transferred across sectors, supporting broader cross-sector innovation.^13,14^

Although drilling fluids represent only a small fraction of the total drilling expenditures, they are critical to the overall success of the operation. Inadequate fluid control in high-risk applications, such as deepwater drilling, can increase operational costs by millions of dollars.^5^ Moreover, drilling fluids influence formation evaluation, affect long-term well productivity, and are increasingly subject to stringent environmental and waste management regulations. These factors make the development, characterization, and formulation of drilling fluids an indispensable aspect of modern drilling operations.^15^

Overbalanced drilling (OBD) is the most employed drilling method, in which the pressure exerted by the drilling fluid inside the wellbore is intentionally maintained above the natural formation pressure. This technique is widely used because it establishes a protective barrier that prevents the influx of formation fluids such as oil, gas, or water while stabilizing the borehole and reducing the likelihood of collapse.^16,17^ However, to sustain this overbalance during drilling operations, the density of the drilling fluid must exceed that corresponding to the formation pore pressure.^16,18^

Maintaining high drilling fluid pressure and density in OBD operations presents several significant drawbacks. The most critical is formation damage, which occurs when the drilling fluid invades and plugs the rock’s pore spaces, thereby reducing permeability and decreasing well productivity.^19^ Other issues include lost circulation, where the fluid infiltrates fractures within the formation, and a reduced rate of penetration (ROP), which increases drilling time and overall costs.^20^ Differential sticking is also a serious concern, as excessive differential pressure can cause the drill string to become stuck against the wellbore wall.^16,21^ These limitations indicate the need for alternative drilling techniques that minimize formation damage and operational risks.

In response to these challenges, the underbalanced drilling (UBD) technique was developed, in which the wellbore pressure is intentionally maintained below the formation pressure. This approach minimizes formation damage by reducing pore blockage while enhancing drilling efficiency through increased rates of penetration and reduced operational time and costs.^22,23^ Additionally, UBD mitigates major risks such as differential sticking and lost circulation, thereby preventing drill string entrapment and fluid losses into formation fractures.^16,24–26^ Because successful UBD operations rely on maintaining reduced wellbore pressures, drilling fluids must be formulated with lower densities, leading to the development of low-density drilling fluids. Figure 2 illustrates the pressure relationship between the drilling fluid and the formation under overbalanced (OBD) and underbalanced (UBD) drilling conditions.

**Figure 2:**
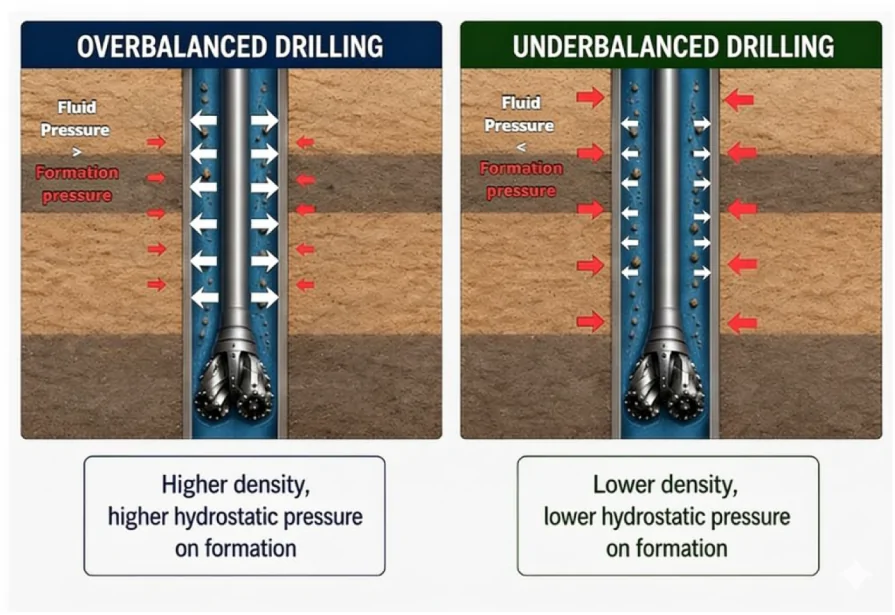
Comparison between OBD and UBD drilling.

Low-density drilling fluid technology is essential to the petroleum industry, as it enables the exploration and development of low-pressure reservoirs and mature fields that would otherwise be inaccessible.^12^ By facilitating enhanced resource recovery, extending well lifespan, and improving overall profitability, this technology significantly broadens the industry’s operational potential. Without such innovation, the sector would remain constrained to overbalanced drilling methods, rendering many reservoirs economically infeasible or technically impossible to exploit.^16,27,28^ This would result in substantial losses of recoverable reserves worldwide and would limit both the growth and adaptability of the industry.^29^

In summary, although lightweight solid additives have been in use since the 1960s for low-density drilling fluid formulations, the range of additives available remains limited. Furthermore, significant gaps persist in our understanding of the individual advantages, limitations, and applications of these additives under varying drilling conditions. This review seeks to overcome current limitations in the field by examining existing trade-offs, identifying critical knowledge gaps, and proposing targeted directions for future innovation. By bringing together literature that is typically dispersed across multiple sources, this review provides a focused synthesis on density-reducing additives, covering studies published over the past decade to support the development of next-generation lightweight drilling fluids.

## 2 Scope and Approach

The technologies employed to reduce the density of drilling fluids are diverse and encompass multiple classes of additives. A comprehensive understanding of the characteristics, specific applications, advantages, and limitations of each additive type is essential in guiding appropriate formulation selection, thereby contributing to safer, more efficient, and economically viable drilling operations within the oil and gas industry.

Therefore, the aim of the present work is to critically examine the evolution of technologies and density-reducing additives used in drilling fluids, with a focus on their main applications, advantages, limitations, costs, safety aspects, and operational performance. In doing so, this review seeks to provide insights that may contribute to the development of more efficient, cost-effective, and safer drilling practices, while offering an updated overview of technologies and additives for density reduction that may serve as a guideline for future applications in the oil and gas industry. For this purpose, a comprehensive search of literature was conducted using the ScienceDirect, Web of Science, and Scopus databases. The keywords used were “low-density drilling fluid additives,” “lightweight drilling fluid additives,” “density reduction drilling fluid additives,” and “underbalanced drilling fluid additives.” A second search was performed using the same keywords, substituting “drilling fluid” with “drilling mud” to broaden the scope of the search. The review considered both experimental and review articles published between 2015 and 2026 within the subject areas of Engineering, Materials Science, and Chemical Engineering, restricted to papers written in English.

In developing this review, the objective was to establish a structured and technically rigorous reference for researchers and industry professionals. The paper is organized to progress from core concepts to advanced developments. It first defines UBD, outlines its operational rationale, and summarizes its historical evolution. Subsequent sections examine the principal low-density technologies introduced over time with an emphasis on their mechanisms, performance characteristics, and operational constraints. The review then synthesizes the most relevant studies published over the past decade, identifying research trends, persistent knowledge gaps, and opportunities for further advancement. This structure provides a clear and integrated perspective on the evolution and application of low-density drilling fluids and supports the translation of current knowledge into improved engineering decisions and operational strategies.

## 3 Overview of Low-Density Drilling Fluids

### 3.1 Historical Timeline

Historical records indicate that the earliest concept of drilling fluids originated in China between 1122 and 250 B.C., and the Chinese employed this technique to soften rock formations and remove debris, using bamboo poles as drilling tools. This method enabled the construction of numerous wells near the Tibetan border, some extending several hundred meters in depth for the extraction of brine, natural gas, and water. However, the concept of drilling fluids did not spread to other parts of the world until the early 19th century.^15,30^

In 1845, French engineer Pierre-Pascal Fauvelle and English inventor Robert Beart independently developed a method for utilizing water in drilling operations. They introduced the concept of pumping water through a hollow drill rod to transport cuttings to the surface—a technique that proved highly effective. Their innovations, which were promptly patented, laid the groundwork for the development of modern drilling rigs and fluid systems.^15,30^

In 1890, the work of M. T. Chapman established the term *drilling mud* within the oil industry. Chapman introduced the use of plastic additives—such as clay, bran, grains, and cement—combined with the water already employed in drilling operations, thereby imparting new functions and properties to the fluid. This modification enabled plastering of the wellbore wall and reduced the likelihood of collapse.^15,30^ This development marked the beginning of systematic research into the behavior of various additives within drilling fluids and the benefits they could provide.

In the mid-1910s, the issue of gas and fluid escaping from wells to the surface led engineers to seek methods for preventing and controlling such leaks. In 1922, drilling supervisor Stroud, working in Louisiana, asserted that the key to successful drilling and well control was directly related to managing gas pressure through the use of heavy mud. To achieve this, he tested additives such as cement, galena, and iron oxide to increase the fluid’s density. By the end of the same year, barite had also begun to be used for the formulation of heavy drilling muds. This milestone established the practice of employing weighting materials to increase fluid density, making density control a fundamental aspect of all drilling operations.^15^

From that period onward, numerous technologies and additives were incorporated into drilling fluids, resulting in new properties, formulations, and performance improvements. A major milestone occurred in the 1950s with the introduction of oil-based drilling fluids, which proved to be more efficient lubricants, thermally stable, and resistant to water invasion—transforming the application dynamics of drilling fluids, particularly in high-temperature environments.^30^ Additionally, the use of oil-based fluids enabled a reduction in overall fluid density, stimulating research into systems with lower densities than conventional drilling fluids.^15^

In the 1960s, the drilling industry began experimenting with gases, foams, and aerated fluids as innovative alternatives to conventional mud systems. During this period, small rigs used for seismic exploration started employing compressed air in areas where water was scarce or where operations were conducted under low-temperature conditions. This practice resulted in higher penetration rates, extended bit life, and, most importantly, more efficient borehole cleaning, which translated into greater productivity compared with traditional drilling muds.^15,30,31^ This development represented another significant milestone in the evolution of low-density drilling fluids, enabling the attainment of densities as low as 0.01 ppg in the case of air drilling and paving the way for subsequent advancements in lightweight drilling formulations.^16^

In 1995, the study conducted by Medley in collaboration with the U.S. Department of Energy^24^ marked a major milestone in the introduction of hollow glass spheres (HGSs) as an innovative additive for reducing drilling fluid density. Although Medley noted that HGS had already been applied in Russia during the 1960s, this was the first published work outside that region to address their use in drilling operations. The incorporation of HGS provided significant advantages over gaseous and aerated fluids, enabling fluid densities below 6 ppg while preserving the benefits of incompressible systems.^24^ This development represented a breakthrough in lightweight drilling fluid technology and laid the foundation for continued research in this field over the following decades.^32–35^

In recent years, the pursuit of more efficient and cost-effective alternatives has driven extensive research on additives designed to reduce the density of conventional drilling fluids. These include low-density polymers, surfactants, foaming agents, nanomaterials, and environmentally friendly compounds, which have been investigated not only for their ability to lower fluid density but also for their ability to improve overall performance in terms of stability, lubrication, and environmental compatibility.^16,30^ Table 1 summarizes the historical evolution of both conventional and low-density drilling fluids and their technological advancements up to the present day.

**Table 1:** Historical Milestones in the Evolution of Drilling Fluids, with Emphasis on Low-Density Systems^a^

**Year**	**Milestone in Drilling Fluid Development**
1122–250 B.C.	Use of water in ancient China to soften rock and transport drilling debris.
1845	Fauvelle (France) and Beart (England) introduced hydraulic circulation using hollow rods.
1890	Chapman proposed the addition of solid additives (e.g., clay, bran, grains, cement) to improve drilling fluid performance.
1922	Stroud demonstrated density control in drilling fluids through the incorporation of heavy mineral additives.
1950	Introduction of oil-based drilling fluids offering improved thermal stability and lubrication.
1960	Development of gaseous, foamed, and aerated drilling fluids.
1995	Medley published the first study outside Russia on the use of HGS for density reduction.
Present	Ongoing research on lightweight additives and advanced low-density drilling fluid formulations.

a Source: refs 15 and 30.

### 3.2 Underbalanced Drilling

As previously discussed, the use of high-density drilling fluids in OBD operations involves significant drawbacks, particularly with respect to formation damage. This phenomenon refers to the reduction in the flow capacity of oil-, gas-, and water-bearing reservoirs. Although it can occur during various stages of well construction and production, drilling is widely recognized as the primary cause, as it represents the first exposure of virgin formations to foreign fluids.^20,21,36–38^ The invasion of these fluids into the rock matrix can obstruct pore spaces around the wellbore, resulting in different types of reservoir impairments.

The main mechanisms of formation damage have been extensively discussed in the literature to elucidate the challenges that they present. Most of these mechanisms are associated with the invasion of drilling fluids into the formation and processes that reduce reservoir permeability and compromise hydrocarbon recovery. Key examples include fine migration, where solid particles are mobilized and block pore throats; external solids invasion, resulting from the entry of weighting materials and other additives present in drilling fluids; phase trapping, in which introduced fluids remain confined within the pore spaces and hinder natural flow; and clay swelling, which occurs when clay minerals react with low-salinity water, expanding and obstructing the pore network.^16,21,39–43^ Given these limitations, the adoption of UBD has gained increasing relevance, as it provides an alternative approach capable of minimizing fluid invasion and substantially reducing formation damage.

The fundamental distinction between the OBD and UBD lies in the pressure exerted on the wellbore wall. UBD is a drilling technique in which the wellbore pressure is intentionally maintained below the exposed formation pressure, thereby minimizing formation damage and enabling the exploitation of low-pressure and depleted reservoirs.^16,44,45^ Achieving and sustaining this pressure differential primarily depends on controlling the density of the drilling fluid; therefore, various methods of density reduction must be investigated to support UBD operations.

## 4 Density Reduction Technologies

The following sections examine both gas-based density reduction technologies and solid additive-based approaches. Gas injection methods are included because they historically represent the first and most widely deployed strategies for achieving underbalanced conditions.

### 4.1 Gas Injection

#### 4.1.1 Air Drilling

Air injection was the first technology developed to reduce drilling fluid density.^15^ This method relies exclusively on compressed air as the circulating medium, with dry air being the most common option, typically delivered through high-capacity compressors. It is considered one of the most economical approaches under favorable operating conditions because it eliminates the need for chemical additives and utilizes a naturally abundant and readily available resource. Air drilling provides very high rates of penetration, and the elevated annular velocities achieved during this process enhance cuttings removal, contributing to longer bit life and more efficient hole cleaning.^15,16,44,46–49^

Air drilling reduces the density by replacing conventional aqueous or oil-based drilling fluids with compressed air. Because air has a much lower density than either water- or oil-based drilling fluids, it exerts a lower hydrostatic pressure on the formation. This density reduction mechanism is not unique to air drilling and is also observed in nitrogen, natural gas, and other gas-based drilling systems. During circulation through the annulus, air density decreases further as the gas expands toward the surface due to pressure reduction. However, because air is highly compressible, substantial variations in density and temperature may occur along the wellbore, requiring careful hydraulic design and operational control.^15,27^

One of the primary drawbacks of air drilling is associated with water influx and wellbore instability. When water-bearing formations are encountered, cuttings can become saturated, adhere together, and are not effectively transported by an air stream. The accumulation of these wet cuttings in the annulus can lead to the formation of a mud ring, obstructing airflow and causing the drill pipe to become stuck.^15,46^ In addition to these water-related challenges, another major drawback arises from the oxygen content of the air, which introduces significant safety and operational risks.

In addition, air drilling poses critical issues due to approximately 21% oxygen present in air. Oxygen is a strong oxidizing agent that, in the presence of moisture, accelerates the corrosion of steel components such as the drill string and casing, generating substantial hidden costs for corrosion control. Moreover, oxygen is one of the three essential elements for combustion; thus, when mixed with hydrocarbons, the air–gas mixture can be ignited by mud rings or localized hot spots from leaks, creating severe fire and explosion hazards that compromise equipment integrity, reduce productivity, and increase operational costs. Finally, although air itself is inexpensive, the need to compress it to pressures exceeding 2,000 psi (≈14,000 kPa) substantially raises overall expenditures.^50^ Consequently, the use of air drilling is recommended only for specific types of formations.^16,44,46^

Because of these limitations, air drilling can be applied only under restricted conditions. It is generally confined to vertical, small-diameter wells in hard and relatively dry formations, where liquid influx is minimal or absent. The technique is also unsuitable for sour-gas reservoirs or environments with elevated downhole fire risks.^16^ In summary, air drilling offers substantial improvements in the penetration rate and hole cleaning efficiency compared with conventional mud systems; however, its applicability remains limited by wellbore instability, corrosion, and safety concerns associated with oxygen and hydrocarbon interactions.

#### 4.1.2 Natural Gas Drilling

Natural gas, primarily composed of methane (CH_4_), can also be employed as a drilling fluid to replace conventional air in underbalanced-drilling operations. Unlike air, natural gas eliminates the presence of oxygen in the wellbore, thereby reducing the risks of explosion and corrosion. Furthermore, when natural gas is already being produced from the formation or when nearby pipelines are available, it becomes an inexpensive and abundant resource, eliminating the need for compressors and reducing the operational costs typically associated with air drilling.^44^ However, despite these benefits, several disadvantages should be carefully evaluated.

The use of natural gas as a drilling fluid presents three primary limitations. First, because it is significantly less dense than air, a larger volumetric flow rate (approximately 20% higher) is required to achieve an equivalent performance. Second, natural gas exhibits high solubility in oil-based drilling fluids. Under elevated wellbore pressures, the gas can completely dissolve into the liquid phase, converting the desired two-phase flow system to a single-phase fluid. This becomes particularly problematic during pressure reduction, as the dissolved gas may suddenly exsolve, forming large, unstable bubbles that compromise drilling stability and well control.^44^ Finally, natural gas poses a significant safety hazard when released at the surface, as it forms a combustible mixture with atmospheric air, thereby increasing the potential for surface fires and explosions and necessitating modifications to standard operating procedures.^51^

#### 4.1.3 Nitrogen Drilling

Nitrogen can also be employed as a drilling fluid and is typically generated either through cryogenic processes or membrane separation techniques. Cryogenic nitrogen, produced at extremely low temperatures (approximately −160 °C), is one of the most widely used gaseous drilling fluids in industry, particularly in onshore operations and regions where liquid nitrogen can be readily transported and supplied. In contrast, the membrane separation method involves passing atmospheric air through a specialized semipermeable membrane that isolates nitrogen from oxygen, carbon dioxide, and water vapor. This type of nitrogen is generally produced on-site and requires a specific sequence of processing and compression equipment for generation.^44,51^ Each production method presents distinct advantages and limitations depending on well conditions and logistical factors associated with the drilling location.

Membrane-based nitrogen production offers several advantages. Similar to natural gas, the risk of explosion or combustion is minimal because of the very low oxygen concentration in the gas mixture. Another key advantage is the excellent compatibility of nitrogen with oil-based drilling fluids. Because nitrogen exhibits very low solubility in oil, it facilitates the formation of stable foamed fluids with reduced density and limited corrosive potential. For this reason, nitrogen is the most widely used gas in foamed drilling systems containing oil-based fluids.^44,52^ Nevertheless, despite these advantages, several disadvantages must also be carefully evaluated before its widespread application.

The membrane-based method of nitrogen generation presents several operational and economic disadvantages. First, it does not fully mitigate corrosion issues, as the nitrogen produced typically contains 4–6% residual oxygen, and removing this oxygen is not economically feasible, given the large gas volumes required for drilling operations. Second, the flare system of the drilling rig may be compromised, allowing hydrocarbon gases to escape unburned into the atmosphere in the presence of nitrogen. Finally, membrane-generated nitrogen substantially increases operational costs due to the system’s relatively low efficiency of approximately 50%, which necessitates supplying nearly twice the volume of air to achieve the desired nitrogen output. These costs are further compounded by the high capital expense of the membrane unit, additional personnel requirements, and mobilization charges.^44^

Similar to the membrane method, the cryogenic nitrogen system offers several advantages. Primarily, this type of nitrogen is completely oxygen-free, effectively eliminating issues associated with corrosion and explosions. Moreover, because liquid nitrogen can be injected at extremely high pressures, it is particularly suitable for coiled tubing operations, where high-pressure delivery is essential.^44^ Nevertheless, as with the membrane-based method discussed previously, certain disadvantages must be carefully considered.

The most significant limitation of the cryogenic nitrogen system is its high cost, primarily driven by the extremely expensive logistics associated with transportation. Although nitrogen gas itself is inexpensive at the manufacturing facility, transporting it in the liquid form requires specialized, proprietary tanks and handling equipment. As a result, the total cost of cryogenic nitrogen can exceed five times that of conventional compressed air and at least double that of membrane-separated nitrogen. Another major concern is boil-off—the vaporization of liquid nitrogen during transit—which is particularly problematic in hot climates or when extended delays occur between tank loading and on-site deployment. In extremely high-temperature regions, such as Venezuela, evaporation losses have been reported to reach up to 50% of the total load. Finally, as with the membrane method, cryogenic nitrogen can affect the rig’s flare system, often resulting in unburned gas being vented into the atmosphere.^44^

In summary, while nitrogen offers excellent stability and safety compared to air and natural gas, its implementation is constrained by high costs, system inefficiencies, and logistical challenges associated with large-scale supply and transportation.

#### 4.1.4 Carbon Dioxide Drilling

Another alternative used in gas-based drilling systems is supercritical carbon dioxide (CO_2_). The primary advantage of CO_2_ is its low cost, as it is more abundant and less expensive than other gases previously discussed while also being more environmentally benign. In addition, CO_2_ is nonflammable and, in its liquid phase, exhibits sufficient density to operate downhole motors and generate adequate torque, which is a challenge where nitrogen alone is less effective. Moreover, as CO_2_ transitions to the gaseous phase in the annular space, it produces a very low bottomhole pressure while maintaining underbalanced drilling characteristics.^16,44,53^ However, the use of CO_2_ also presents several important disadvantages that must be carefully considered.

The main limitation of the CO_2_ drilling fluids is corrosion. When CO_2_ combines with water, it forms carbonic acid (H_2_CO_3_), which lowers the pH of the fluid and significantly increases its corrosion. This acid reacts with iron and cement, generating byproducts that degrade structural well components, including metal hardware and cement sheaths. In addition, CO_2_ is odorless and colorless and can be toxic at concentrations exceeding 5% by volume, which poses serious safety risks. It also exhibits high solubility in water (approximately 0.9:1 by volume at standard conditions), leading to dissolution into other fluids within the well, complicating fluid maintenance and monitoring.^44^

### 4.2 Mist Drilling

Another low-density technique used in underbalanced drilling operations is *mist drilling*. This approach involves introducing a small amount of water, along with a foaming agent, into the gas stream. It is most commonly applied when drilling through formations that produce moderate water influxes (10–100 bbl/h), which restrict the use of dry-air drilling. The foaming agent is typically added at very low concentrations (0.1–0.25% by volume), while the total water content generally remains below 2.5%.^16,51^

Unlike conventional drilling fluids, in which the liquid phase is dominant, mist systems are composed primarily of a gas. As a result, they exhibit a low overall density because the gas phase is much less dense than the liquid phase. Mist density can be adjusted by controlling the gas-to-liquid ratio; as the gas fraction increases, the average density decreases, allowing underbalanced conditions to be achieved. Similar to air drilling, gas compressibility causes mist density to vary with depth and pressure along the wellbore.^17,44^ Therefore, as with other low-density drilling methods, both the advantages and the limitations of mist drilling must be carefully considered.

The primary advantages of mist drilling are its high tolerance to water influx and its ability to prevent *mud ring* formation, which is a common issue in dry-air drilling. This capability is critical for minimizing the risk of the pipe sticking. Additionally, mist drilling provides superior wellbore cleaning efficiency compared with dry-air systems and reduces the likelihood of downhole fires.^16,51^ However, several disadvantages must be addressed.

On the other hand, the major limitation is the requirement for higher injection volumes—approximately 40% greater than in air drilling—and consequently higher pressures, about 100 psi higher, which increase compressor demand and fuel consumption. Moreover, because water is present in the circulation system, a treatment or disposal system is required to manage wastewater volumes ranging from 200 to 500 bbl/day, further increasing operational costs. The presence of water can also destabilize water-sensitive formations (e.g., shales) through swelling and collapse. Finally, the combined presence of oxygen and water intensifies corrosion, leading to equipment degradation and necessitating the use of costly corrosion inhibitors.^16,51^

### 4.3 Aerated Drilling

Another technology applied in UBD is *aerated drilling*, which represents an intermediate approach between dense liquid systems and light-gas systems. It involves injecting compressed gas into a liquid drilling fluid to reduce its density while largely retaining its original fluid properties. Various liquids can serve as the base fluid, while gases such as air, nitrogen, or natural gas are commonly used.^16,51^ This technique offers significant performance advantages but also presents operational limitations.

In aerated drilling, density reduction is achieved by injecting gases, such as air or nitrogen, into a continuously circulating liquid drilling fluid. Unlike air- or mist-drilling, in which the gaseous phase predominates, aerated systems consist of a continuous liquid phase containing dispersed gas bubbles. The addition of a low-density gas phase reduces the average density of the circulating fluid, thereby decreasing the hydrostatic pressure exerted on the formation. In this case, density reduction depends mainly on the gas volume fraction, bubble distribution, and downhole pressure conditions. As the fluid flows upward and pressure decreases, gas expansion increases the gas volume fraction, further altering the density profile along the wellbore.^27,54^

Building on the advantages of UBDs already discussed, aerated drilling provides two notable benefits. First, it exhibits a much higher tolerance to fluid influx than most other UBD methods. Second, it is not temperature-sensitive and has no strict operational temperature limits, providing substantial flexibility.^51^ Additionally, further advantages have been reported, including highly efficient cuttings transport and excellent wellbore cleaning.^16^ Because of the reduced fluid density, aerated drilling systems also help mitigate the risk of differential sticking of the drill string.

However, this technique has several pressure-related limitations. Since the gas phase is compressible, pressure changes alter both the quality and proportion of the gas–liquid mixture, thereby affecting overall fluid properties. Another major limitation is the occurrence of pressure surges, caused by large gas clusters rising rapidly to the surface and generating severe pressure fluctuations that may compromise wellbore stability and damage the formation. Managing the gas–liquid ratio is also critical: insufficient gas increases bottomhole pressure and may cause the system to become overbalanced, whereas excessive gas can lead to slugging. Additional challenges include corrosion, increased friction factors (resulting in higher torque and drag), drill-string vibration (as the aerated fluid does not fully support the pipe), and elevated operational costs.^16,51^

### 4.4 Foam Drilling

The next technological advancement after aerated drilling fluids was the incorporation of surfactants, leading to the development of *foam drilling fluids*. The addition of surfactants stabilizes the liquid films surrounding gas bubbles, promoting foam persistence and reducing bubble coalescence. Compared with conventional aerated fluids, foam systems exhibit greater homogeneity and improved stability under a wide range of drilling conditions. However, foam stability is strongly influenced by operational parameters such as temperature, pressure, fluid composition, and contamination and may decrease under severe downhole conditions, particularly in high-temperature environments.^55–73^ Air is most commonly used as the gas phase, although nitrogen and natural gas are also applied in practice. For the liquid phase, fresh water is typically preferred because of its low cost, but the resulting foam quality can be limited. Brines may be used as an alternative when combined with suitable surfactants, whereas oil-based liquids are generally avoided because they rapidly destabilize the foam.^16,44,51^

Density reduction in foam drilling is primarily achieved by incorporating a high fraction of gas into the fluid and replacing a substantial portion of the denser liquid phase. This decreases the average fluid density and, consequently, reduces the hydrostatic pressure exerted on the bottom of the well. This reduction in bottom-hole pressure is the fundamental mechanism that enables underbalanced drilling conditions.^16,74^

This system offers several advantages. Pressure surges common in aerated fluids are greatly reduced as the stabilized foam minimizes fluctuations, leading to safer and more efficient operations with improved reservoir protection. Foam drilling also enables extremely low bottomhole pressures and provides excellent cuttings lifting capacity, ensuring efficient removal of debris even at low annular velocities. Additionally, this method can increase the rate of penetration and nearly eliminate drilling fluid losses.^44^ Another significant advantage is operational flexibility, since foam quality, foaming agent type, and stabilizer selection can be adjusted to enhance performance and adapt to varying drilling conditions.^16,75^

However, several disadvantages must also be considered, primarily related to cost and foam stability. Operating costs are comparable to other UBD techniques, as similar equipment—compressors and separators—is required, but the expense of foaming agents, stabilizers, and corrosion inhibitors is high. In wells with temperatures above 100 °C, total costs can exceed USD 2,000 per day.^44^ Foam stability is another major concern, as it tends to collapse or migrate in high-temperature wells, especially during operational maneuvers. Additionally, foam can be degraded when contaminated at the surface by light oil, hot brine, or sour water, which act as defoaming agents. Contamination by acidic (pH < 5) or ion-rich water further increases maintenance costs, requiring higher concentrations of the foaming agent or complete fluid replacement.

To overcome foam-stability challenges under severe reservoir conditions, recent technological advances have focused on nanostructured materials, polymers, and environmentally friendly additives. One example is the development of Pickering foams stabilized by nanoparticles and cellulose nanofibers (CNFs). These nanofibers can adsorb irreversibly at gas–liquid interfaces, forming a robust mechanical barrier that prevents bubble coalescence and enhances thermal resistance and stability under high-salinity conditions.^74^ Another innovative approach involves salt-responsive polymers, such as HPAACM copolymers.^76^ Hydrophobic modifications allow these polymers to form three-dimensional viscoelastic networks under the high-temperature and high-salinity conditions typically found in reservoirs, in contrast to the degradation commonly observed in conventional polymers. Recent studies have also explored sustainable foam formulations based on environmentally friendly components, including polyanionic cellulose, starch, and surfactants, to produce stable eco-friendly foams under mildly alkaline conditions.^64^

Field-scale foam drilling still faces several practical constraints and financial barriers. Scaling up foam operations requires specialized surface control and snubbing equipment to safely manage multiphase fluid recovery and wellbore pressure control, which increases operational complexity and cost. From an economic perspective, continuous nitrogen injection can also substantially increase operating expenses. In deep and/or high-temperature, high-pressure wells, conventional additives and polymers are highly susceptible to thermal and mechanical degradation, which can increase interfacial tension, reduce viscosity, and ultimately lead to foam failure. Therefore, the economic viability of foam drilling depends on balancing the higher costs associated with equipment, gas generation, and specialty chemicals against the benefits of faster drilling, reduced formation damage, and improved long-term well productivity.^16,55,74^

Postoperation, foam treatment and disposal also pose environmental and waste-management challenges.^16,44,51^ Under standard temperature and pressure conditions, some foamed-fluid formulations may exhibit high kinetic stability, delaying bubble rupture and the separation of liquid and gas phases; this can create difficulties in gas–oil separation processes.^77^ As a result, treatment and disposal become more complex because residual foam can hinder solid–liquid separation, reduce the efficiency of wastewater-treatment equipment, and increase effluent-conditioning time. In such cases, special defoamers and additional measures, such as pH adjustment, may be required to promote controlled foam collapse. Therefore, foam stability must be carefully controlled to balance operational performance with the feasibility of downstream processing and waste management.^78,79^

### 4.5 Engineered Lightweight Additives

Another approach developed to reduce overall drilling fluid density is the incorporation of low-density solid additives. The concept was first formally proposed in the Western literature in 1995, when the use of HGS in drilling fluids was proposed.^24^ The objective was to eliminate the disadvantages associated with gas compressibility by formulating a liquid-based, incompressible, low-density system that could retain the benefits of UBD while minimizing its limitations.^24^ Building on this foundation, subsequent studies have investigated various low-density materials for similar applications.

In HGS-based drilling fluids or in any fluid system containing hollow particulates, density reduction is achieved by incorporating low-density hollow particles into the drilling fluid. Hollow glass spheres consist of internal cavities enclosed by thin glass shells, providing a relatively large occupied volume with minimal mass addition. As a result, their incorporation reduces the average fluid density while maintaining a solid particulate phase in the formulation. The density reduction mechanism is therefore primarily associated with the hollow structure of the particles, which contributes substantially to the volume without significantly increasing the total mass of the system. In suspension, the low density of HGS particles may also reduce their tendency to settle, helping maintain a more homogeneous dispersion when supported by adequate fluid rheology. However, the performance of HGS-based drilling fluids depends not only on microsphere density but also on concentration, particle size distribution, packing behavior, and structural integrity under pressure. Interactions between HGS particles and other fluid components, such as polymers, clays, and weighting agents, can also influence suspension stability, rheological behavior, and particle dispersion. Therefore, the effectiveness of HGS-based density reduction depends on both microsphere properties and compatibility with the overall drilling fluid formulation.^32,80^

More recent research has focused on natural and biodegradable alternatives. Adewole and Najimu^81,82^ demonstrated that date pits are an effective additive for reducing density in aqueous fluids while also being environmentally friendly and biodegradable.^81,82^ Similarly, *Green Olive Pits* have been investigated as sustainable density-reducing agents,^83^ and mandarin peel powder has shown comparable results.^84^ These studies illustrate that the development of low-density additives remains an active research area with substantial potential for innovation.

The use of low-density solids in drilling fluids shows strong promise for the future of UBD. Such systems combine the advantages of incompressible fluids with the performance benefits of lightweight formulations, avoiding the major drawbacks of gas-based systems—such as compressibility, corrosion, complex pressure management, safety risks, and high operational costs.^24^ Although research is still limited, current findings suggest that this technology could become one of the most promising directions for future UBD advancements.

### 4.6 Operational Applicability, Fluid Formulation, and Additive Selection

To clarify the operational scope of UBD techniques, a synthesis of the major UBD technologies and their most suitable geological settings and field applications was developed. The resulting summary is presented in Table 2.

**Table 2:** Operational Applicability of Compressible Underbalanced Drilling Technologies Based on Density-Reduction Mechanisms

**UBD Technology**	**Ideal Formations**	**Reference**
Air Drilling	Hard, dry, low-pressure formations and vertical, small-diameter wells	^16^
Mist Drilling	Formations initially suitable for dry-air drilling but affected by moderate water influx	^44^
Foam Drilling	Unconsolidated or fractured formations requiring borehole support	^51^
Aerated Drilling	Fractured limestone formations and water-sensitive shales	^44^
Nitrogen Drilling	Wells where nitrogen generation and transportation logistics are economically feasible and where high pressures are required	^16^
CO_2_ Drilling	Wells with short operating cycles that leverage the high-density/low-density phase change	^44^
Natural Gas Drilling	Low-pressure reservoirs with a nearby natural gas pipeline	^44^

Geological, operational, and economic conditions strongly influence the selection of suitable low-density drilling technologies. Techniques incorporating lightweight gas phases are generally more appropriate in environments where formation instability or uncontrolled influx may threaten well integrity and where precise pressure management is essential. Conversely, methods that rely on a continuous supply of CO_2_ or nitrogen may be less practical in remote areas or in projects with limited budgets. In such cases, solutions that utilize readily available resources—such as natural gas from nearby infrastructure—can substantially reduce operating costs. Consequently, technology selection must balance geological compatibility with constraints related to equipment availability, supply chain complexity, and the operational feasibility of maintaining low-density conditions throughout the drilling program.^23,44,51^

In addition to these constraints, the achievable density range of each method is a key determinant of the selection process. As shown in Table 3, systems such as air, mist, foam, aerated fluids, and other lightweight formulations exhibit markedly different density profiles, which directly influence pressure control and wellbore stability. Aligning reservoir requirements with the capacity of each fluid system to sustain underbalanced conditions is therefore essential.^44^

**Table 3:** Typical Density Ranges and Gas–Liquid Ratios for UBD Fluid Systems^a^

**Fluid type**	**Density (ppg)**	**Gas ratio**
Air/gas	0.01–0.1	>99%
Mist	0.1–0.3	96–99%
Foam	0.3–7	55–99%
Aerated fluids	4–7	<55%
Lightweight fluids	5–8	None

a Source: adapted from ref 45.

The drilling fluid formulation relies on a wide range of additives designed to perform specific functions, including density control, rheology modification, fluid-loss reduction, wellbore stabilization, and lost-circulation prevention. Over the years, several commercial additives based on different materials and mechanisms have been used by the oil and gas industry to address challenging drilling conditions. These additives include lightweight materials, viscosifiers, fluid loss control agents, bridging agents, and sealants. Examples of commercially available additives are presented in Table 4.

**Table 4:** Commercial Additives Commonly Used in Drilling Fluid Formulations for Oil and Gas Operations

**Product**	**Manufacturer**	**Material**	**Function**
3 M HGS	3M	Hollow glass microspheres	Density reduction
Sphericel	Potters Industries	Hollow glass spheres	Density reduction
Expancel	Nouryon	Polymeric microspheres	Density reduction
Dualite	Chase Corporation	Polymeric microspheres	Density reduction
Fillite	LKAB Minerals	Cenospheres	Density reduction
BARAZAN D Plus	Halliburton	Xanthan gum biopolymer	Viscosifier and suspension agent
DUOTEC NS	SLB	Modified starch polymer	Fluid loss reducer
Hydro-Plug	Halliburton	Cellulosic lost-circulation material	Fracture sealing and loss prevention
Therma-Chek	Halliburton	Modified starch	High-temperature fluid loss control
PAC-LV	Several suppliers	Low-viscosity polyanionic cellulose	Fluid loss reducer
PAC-R	Several suppliers	Regular polyanionic cellulose	Fluid loss control and viscosity enhancement
Calcium Carbonate	Several suppliers	Sized carbonate particles	Bridging, reservoir protection, and temporary plugging

### 4.7 Field Applications and Operational Challenges

Field applications provide valuable insights into the actual performance, advantages, and limitations of underbalanced drilling technologies. Although laboratory tests and mathematical models are essential for understanding drilling fluid behavior, field applications provide practical evidence of the effectiveness of these technologies in addressing challenges such as lost circulation, formation damage, pressure control, and drilling efficiency. Several cases reported in the literature have demonstrated the successful use of air drilling, nitrogen drilling, aerated fluids, foams, and lightweight drilling fluids under different geological conditions, including depleted reservoirs, highly fractured formations, and pressure-sensitive intervals. The following cases summarize the selected field experiences.

In air and gas drilling operations, as shown by case studies in the Marcellus Shale and several Brazilian basins, operators frequently encounter severe operational limitations associated with the absence of a damping fluid column. The lack of hydrostatic support can generate strong downhole shocks and drillstring vibrations, leading to mechanical washouts and severe equipment fatigue. Air drilling also has a limited ability to handle significant formation fluid influxes, which can compromise wellbore stability, promote mud-ring formation, impair hole cleaning, and increase the risk of stuck pipe. Scale-up remains challenging when air drilling is extended from simple vertical sections to complex directional wells and offshore environments. Such applications require highly specialized and ruggedized bottom-hole assemblies (BHAs), including reengineered mud motors, vibration dampeners, and electromagnetic MWD tools capable of operating under harsh, undamped conditions. From an economic perspective, the feasibility of air drilling is strongly dependent on the operational context. In the Marcellus Shale, pad drilling has demonstrated significant economic benefits by eliminating drilling fluid costs, reducing cuttings disposal requirements, and substantially increasing ROP. However, in remote or logistically complex regions, such as the Amazon Basin, the high costs of transporting and maintaining specialized surface equipment, including large compressors and boosters, can offset the financial benefits of faster drilling.^48,85^

A relevant example of nitrogen-assisted drilling is the highly fractured Parsi Field in Iran, where drilling was performed by using a combination of crude oil and nitrogen. The use of nitrogen with crude oil helped mitigate operational complications associated with additive degradation and fluid instability under reservoir pressure and temperature conditions. Although this method eliminated circulation losses and improved the ROP in the Parsi well, field-scale implementation still involved important challenges. The main limitation was the need for complex surface equipment for multiphase fluid separation and pressure control, including snubbing equipment to safely strip the drillstring. Moreover, although nitrogen reduces the downhole fire and explosion risks associated with air drilling, continuous generation and injection of large nitrogen volumes, approximately 1500 scfm in the Parsi Field, significantly increases operational costs due to additional equipment requirements.^28^

Field applications have also demonstrated the effectiveness of aerated drilling in depleted reservoirs. Examples include depleted coal seam gas reservoirs in the Surat Basin, Australia, where conventional mud weights between 6.0 and 9.0 ppg caused significant losses, and depleted offshore formations in Angola, where formation pressures were equivalent to a fluid density of approximately 5.8 ppg, at a true vertical depth of about 3600 m. In these cases, aerated drilling helped operators maintain appropriate pressure control, with an overbalance of 15 to 20 bar and fluid losses below 1 m^3^/h. Reported benefits included reduced circulation losses, prevention of differential sticking, improved reservoir protection, enhanced drilling performance, and adequate cuttings transport. However, aerated drilling requires precise control of gas injection and the bottom-hole pressure. Operational challenges include multiphase flow instability, surface gas handling, wellbore stability under reduced pressure, slugging, downhole fire hazards, and increased operational complexity. Therefore, aerated drilling is particularly suitable for depleted formations requiring moderate density reduction and controlled pressure balance.^86,87^

Foam drilling fluids have also been successfully applied worldwide, especially in low-pressure, depleted, damage-sensitive, and highly fractured formations.^16^ One example is the Qadirpur limestone formation in Pakistan, where nitrified foam helped address fluid-loss problems, minimize formation damage, achieve a zero skin factor, and improve gas production compared with nearby wells.^88^ Another successful application was reported in the Hassi Messaoud Field in Algeria, where underbalanced drilling with foam enabled faster and less damaging drilling operations. In this case, oil production rates were nearly 4.5 times higher than those obtained using conventional methods, allowing approximately 40% of drilling costs to be recovered.^89^ Foams have also been used in low-pressure coalbed methane (CBM) formations.^55^

Field applications of HGSs have demonstrated their effectiveness in both high-pressure and depleted reservoir environments. In deep, high-pressure formations, such as the Montney Formation and the Cretaceous bedrock in Canada, where hydrostatic pressures ranged from 2000 to 3300 psi, the incorporation of HGSs reduced the mud density to values between 6.88 and 8.52 ppg without destabilizing the drilling fluid under wellbore conditions. HGS technology helped reduce nonproductive time, increase ROP by approximately 36%, and minimize fluid losses compared with adjacent wells.^90^ Similar results were reported for the Miocene L-III limestone formation in the Mumbai High Field, India, where formation pressures corresponded to an equivalent mud weight of 6.5 ppg. In this case, HGS-based fluids maintained mud densities between 7.2 and 7.9 ppg, reducing fluid losses from more than 100 barrels per hour with conventional drilling fluids to 6–8 barrels per hour, while also improving reservoir productivity.^91^

Overall, field applications indicate that lightweight and underbalanced drilling technologies can provide important advantages in terms of pressure control, formation protection, reduced fluid loss, and improved drilling efficiency. However, their successful implementation depends on careful consideration of operational complexity, surface equipment requirements, pressure control, fluid stability, additive durability, and economic viability. For HGS-based fluids, in particular, efficient application depends on the mechanical strength of the particles under high-pressure and high-shear conditions since particle breakage can compromise density reduction and alter fluid properties. In addition, the relatively high cost of advanced lightweight additives remains a limiting factor for widespread field deployment.^90,91^

Notably, most documented field applications are still associated with conventional underbalanced drilling fluids, such as air-, gas-, aerated-, and foam-based drilling systems, while field validation of emerging density-reducing additives, including polymeric hollow microspheres, ultralightweight solids, and other engineered low-density materials, remains limited.

## 5 Critical Review of Density-Reducing Additives

Several review articles have addressed topics related to lightweight drilling technologies, including underbalanced drilling operations, drilling fluid design, and hollow microsphere applications. However, these studies generally focus on specific technologies, materials, or operational aspects, providing only a partial perspective of density-reduction strategies. In contrast, the present review adopts a broader approach by integrating compressible and incompressible low-density systems, examining both established and emerging density-reducing additives, and discussing laboratory investigations, field applications, commercial technologies, and future research directions. To better illustrate the scope and novelty of this work in relation to previously published reviews, a comparative analysis is presented in Table 5.

**Table 5:** Comparative Analysis of Previous Review Articles and the Contributions of the Present Review

**Review**	**Scope**	**Contribution**
^92^	Depleted zone drilling	Comprehensive review of drilling fluid designs and mitigation strategies for depleted-zone drilling, focusing on lost circulation, pipe sticking, and formation damage.
^93^	Uses and properties of Hollow glass microspheres.	Review of the properties, production technologies, and industrial applications of hollow glass microspheres.
^55^	Foam-based fluids for oil and gas reservoirs	Comprehensive review of foam fracturing fluids, covering foam generation mechanisms, stability, additives, field applications, and performance relative to conventional water-based fracturing fluids.
^94^	Underbalanced drilling for depleted reservoirs.	Operational strategies for minimizing formation damage and achieving drilling objectives in highly depleted reservoirs
^32^	Overview of HGS in drilling fluids	Compiles and evaluates studies on the use of hollow glass spheres for drilling fluid density reduction
^54^	Low-density drill-in fluids for depleted reservoirs	Reviews and compares technologies for drilling depleted reservoirs, including their applications, advantages, and limitations.
^16^	Foam drilling fluids and underbalanced drilling	Reviews foam-based UBD systems, covering fluid properties, operational applications, advantages, and limitations.
This work	Density reduction technologies for drilling fluids	Provides a critical assessment of density reducing additives for drilling fluids, identifying research gaps, comparing existing technologies, and proposing future research directions.

Applying the search methodology described in Section 2, a total of 54 articles were retrieved from the databases Scopus, Web of Science, and ScienceDirect after removing duplicates. Following the initial screening, a detailed full-text review was conducted to assess the relevance of each study to the focus of this perspective. This process resulted in the exclusion of 26 articles, leaving 28 eligible publications for this review. Several factors explain the relatively small number of studies identified and the high exclusion rate.

The subject discussed here remains a relatively new and underexplored area of research, even after consulting three major databases. Many excluded studies mentioned “low-density additives” but evaluated materials with normal or even high densities (e.g., bentonite, perlite), making them unsuitable for inclusion. A significant portion of the excluded works also examined low-density systems but focused on other parameters rather than density reduction, often without specifying the density range used in UBD applications. Additional exclusions occurred due to restricted database access or because the studies were outside the defined scope of this review.

The 28 retained articles were considered highly relevant and consistent with the objectives of this review, prioritizing quality over volume. Among these, 23 were experimental investigations and 5 were review studies. Experimental results were evaluated through graphical analysis, and summary tables were compiled to consolidate the principal findings and trends reported in the literature. Figure 3 presents the distribution of studies according to the type of base fluid employed (water- or oil-based).

**Figure 3:**
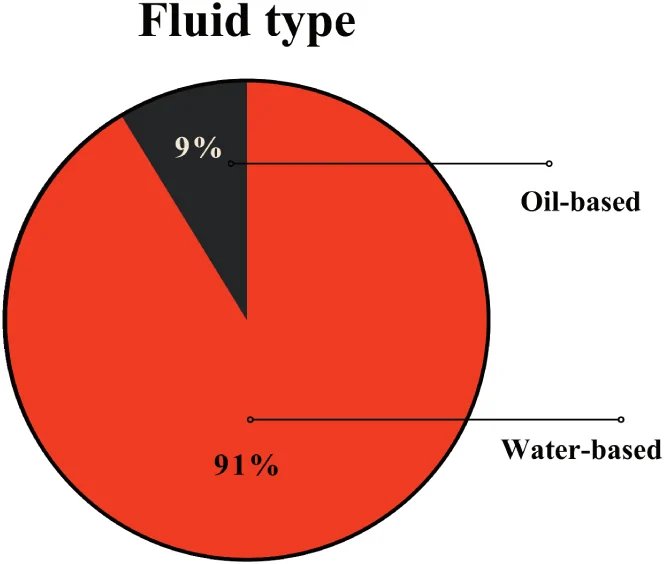
Distribution of drilling fluid types reported in studies investigating low-density drilling fluid additives.

Figure 3 shows that approximately 91% of the reported formulations are water-based, whereas only 9% are oil-based. This predominance likely reflects the industry’s transition toward more environmentally sustainable drilling systems. Water-based muds exhibit lower toxicity, simpler waste disposal, and greater compatibility with eco-friendly additives, aligning with current environmental and economic demands for “green” drilling technologies. However, water-based fluids are inherently denser than oil-based systems because of the higher density of water. Therefore, further research on oil-based low-density fluids is needed, particularly on developing environmentally benign formulations that combine low density with ecological compatibility.

Oil-based drilling fluids exhibit a number of significant drawbacks that call for future research efforts. These systems are associated with higher operational costs due to the use of oil as the continuous phase, the increased complexity of formulation, and the need to maintain emulsion stability. In addition, these fluids raise significant environmental concerns as drill cuttings and waste fluids may contain toxic hydrocarbons, demanding treatment and disposal procedures. Regulatory constraints, particularly in offshore operations, further limit their application. From a technical perspective, oil-based fluids may also induce formation damage through stable emulsions and pore blockage depending on the interaction with reservoir fluids. These limitations indicate the need for continued research into improved formulations and more sustainable drilling fluid systems.^95–97^

Figure 4 shows the distribution of studies according to the type of additive used to achieve a density reduction.

**Figure 4:**
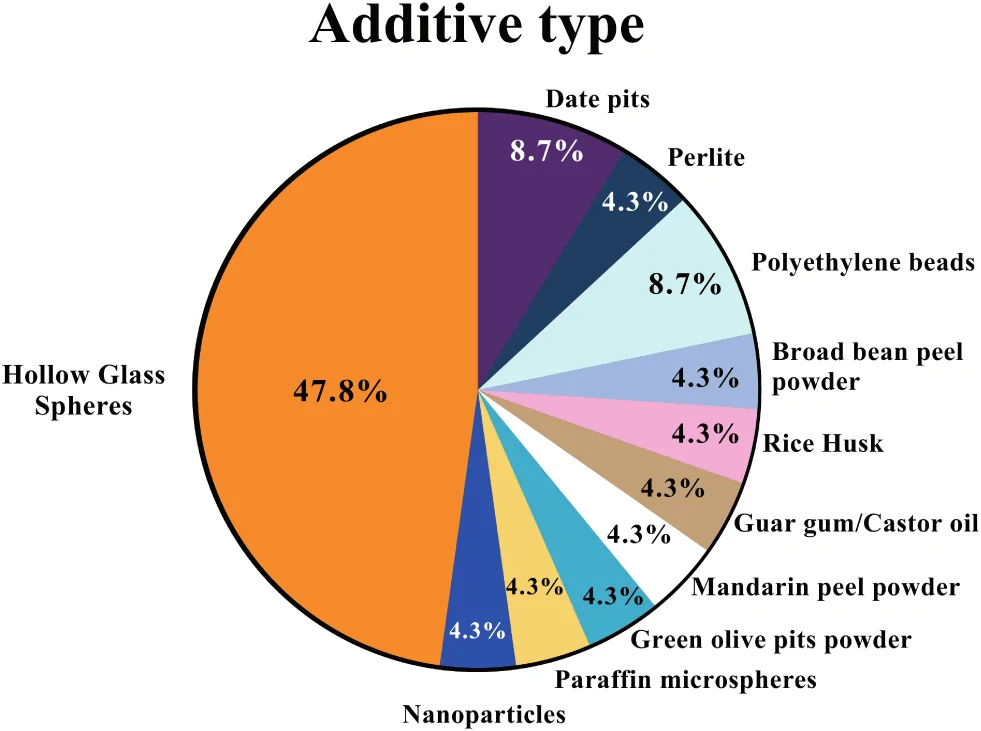
Percentage distribution of the type of additives employed in low-density drilling fluids.

Figure 4 illustrates that hollow glass spheres remain the most widely studied additives for density reduction. Since their first reported use in 1995, HGSs have been successfully incorporated into numerous drilling fluid systems and field applications. Their low density and high compressive strength make them highly effective in reducing fluid weight while maintaining favorable rheological properties and stability. Other additives, though less common, include biobased or waste materials such as *broad bean peel powder*, *rice husk*, *mandarin peel powder*, and *guar gum/castor oil* composites. The diversity of these materials is associated with the growing interest in sustainable, low-cost alternatives to synthetic microspheres, which, despite their excellent performance, remain relatively expensive.

On the basis of the literature analysis and graphical results, three main trends can be identified: (I) the predominance of water-based fluids over oil-based alternatives, (II) the widespread use of solid and biodegradable additives for density reduction, and (III) the dominance of hollow glass spheres (HGSs) as the most common additive used to reduce drilling fluid density.

Figure 5 presents the types of additives identified in the literature, together with their corresponding density values.

**Figure 5:**
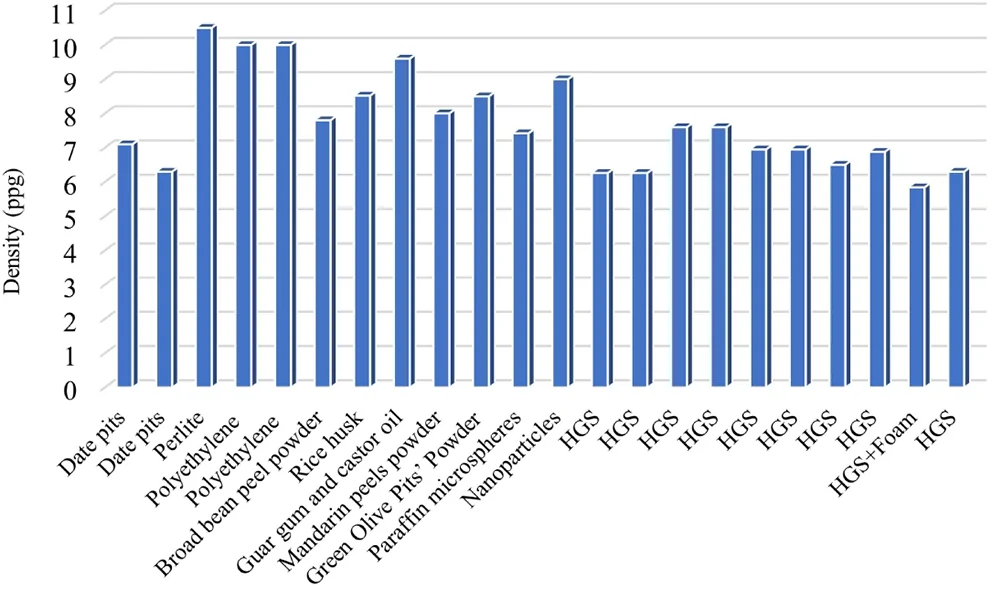
Density of drilling fluids formulated with different types of additives.

Figure 6 shows the percentage of density reduction reported in the reviewed studies for each type of additive.

**Figure 6:**
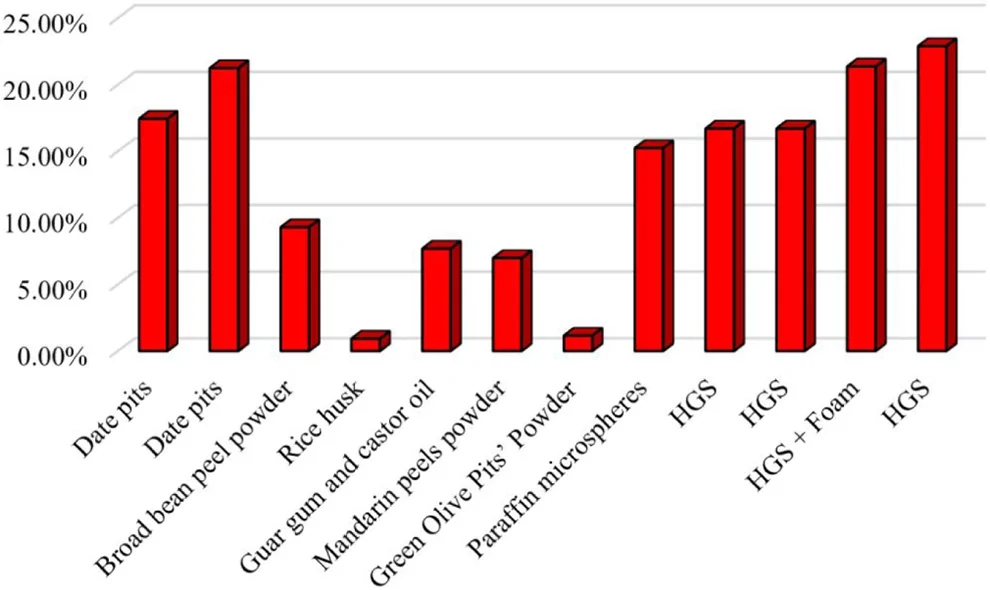
Percentage of reduction for different types of additives used in drilling fluid formulation.

Although the reviewed studies focused on low-density drilling fluid additives, the resulting fluid densities varied markedly. Some additives, such as rice husk and green olive pit powder, exhibited negligible influence on density, suggesting that their primary contribution is linked to enhancements in rheological or thermal properties rather than direct density reduction.^83,98^ In contrast, several materials demonstrated substantial density-lowering capability, including hollow glass spheres (HGSs), HGS combined with foam, and date pits, yielding fluid densities on the order of 6 ppg. The lowest density reported (5.84 ppg) was achieved using a formulation that incorporated HGS and foaming agents to produce an ultralow-density foamed fluid. The greatest density reductions—exceeding 20%—were observed with systems containing HGS and date pits.

Current literature provides strong support for the use of water-based, environmentally responsible drilling fluids formulated with low-density additives—particularly HGS and biogenic powders. These systems are capable of achieving substantial density reductions while maintaining acceptable mechanical and rheological performance. Although the reported results are promising, this research area remains relatively young and only moderately explored. The limited number of publications, combined with the diversity of emerging additive materials, indicates considerable potential for future innovation and continued development in low-density drilling fluid technologies.

The geographical distribution of the reviewed studies indicates that research on low-density drilling fluids remains concentrated in a few key regions with limited global coverage, as shown in Figure 7.

**Figure 7:**
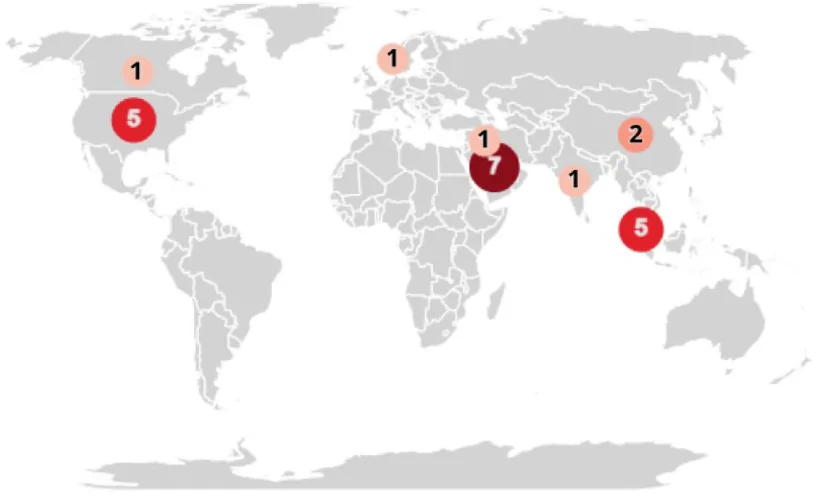
Number of published papers considering density-reducing drilling-fluid additives around the world: Saudi Arabia (7), Malaysia (5), the USA (5), China (2), Iraq (1), India (1), Norway (1), and Canada (1).

Most investigations originate from oil-producing and energy-focused countries, particularly those with strong industrial and academic engagement in drilling optimization and sustainable technologies. Saudi Arabia leads in publication volume, followed by Malaysia and the United States—nations characterized by significant investment in energy research, growing emphasis on environmentally sustainable drilling operations, and well-established academic infrastructure. Overall, the current geographical trend suggests that research on low-density drilling fluids is still in its early developmental stage, with considerable potential for expansion. Therefore, broader international collaboration and continued research efforts are needed to explore new opportunities in this emerging field. Table 6 summarizes the experimental studies identified in the literature.

**Table 6:** Summary of Experimental Studies on Density-Reducing Drilling Fluid Additives (2015–2026)

**Fluid type**	**Additive type**	**Objective**	**Density reduction**	**Final Density (ppg)**	**Refer.**
Bentonite water based	Date pits	Development of a multifunctional, green-based additive derived from date pits	17.44%	7.1	^82^
Bentonite water based	Date pits	Development of a new, lightweight additive derived from date pits	21.25%	6.3	^81^
Calcite water based	Perlite	Potential of perlite as an additive for low-density drilling	Negligible	10.5	^99^
Bentonite water based	Polyethylene beads	Use of polyethylene beads to improve hole cleaning	Not measured	10	^100^
Bentonite water based	Polyethylene beads	Use of polyethylene beads to improve cuttings transportation	Not measured	10	^101^
Bentonite water based	Broad bean peel powder	Use of BBPP as an additive to improve performance	9.3%	7.8	^102^
Water based	Rice husk	Formulate a nondamage fluid with rice husk	0.93%	8.52	^98^
Water based	Guar gum and castor oil	Rheological performance of guar gum and castor oil as additives	7.69%	9.6	^103^
Water based	Mandarin peels powder	MPP to substitute the conventional additives	6.98%	8	^84^
Water based	Green Olive Pits’ Powder	GOPP to substitute the conventional additives	1.16%	8.5	^83^
Not specified	Paraffin microspheres	Synthesis of paraffin microspheres for use as additive	15.24%	7.42	^104^
Water based	Nanosilica, nanoaluminum and nanotitanium	Investigation of rheological properties using nanoparticles	None	9.0	^105^
Water based	Hollow-glass spheres	Lightweight biopolymer drilling fluid for underbalanced drilling	16.71%	6.26	^106^
Water based	Hollow-glass spheres	Lightweight biopolymer drilling fluid for underbalanced drilling	16.71%	6.26	^107^
Water based	Hollow-glass spheres	Stability of Hollow Glass Spheres in Diverse pH’s	Negligible	7.6	^108^
Water based	Hollow-glass spheres	Stability of Hollow Glass Spheres in cement slurry	Not measured	7.6	^109^
Water based	Hollow-glass spheres	Reliability of hollow glass spheres under physical impact forces	Not measured	Not reported	^32^
Water based	Hollow-glass spheres	Durability of hollow glass spheres under physical impact forces	Not measured	6.95	^110^
Water based	Hollow-glass spheres	Investigate the survival rate of HGS	Not measured	Not reported	^111^
Oil based	Hollow-glass spheres	Use of HGS in depleted limestone reservoirs in the Valhall field	Not measured	6.5	^112^
Oil based	Hollow-glass spheres	Use of HGS as a nitrogen substitute in Kakwa field, Canada	Not measured	6.88	^34^
Water based	Hollow-glass spheres + Foaming agent	Formulate an ultralow-density drilling fluid with HGS + foam	21.37%	5.84	^33^
Water based	Hollow-glass spheres	Density and Hydraulic Drag Reduction with HGS	22.9%	6.3	^80^

### 5.1 Date Pits

One of the materials currently under investigation as a low-density additive is the *date pit*. Adewole et al.^81^ evaluated its applicability by first removing the outer layer through micronized washing and grinding the pits into fine particles. The additive was prepared via a microwave-assisted liquefaction process using a mixture of 1 g of date pit, 10 g of ethylene glycol, and 0.2 g of sulfuric acid. After chemical treatment, the product was incorporated into a simple water-based drilling fluid composed of 350 mL of tap water and 20 g of bentonite. The additive was tested at concentrations of 2, 5, and 15 mL, with the optimal result observed at 5 mL, reducing fluid density from 8.0 to 6.3 ppg—a decrease of approximately 21%.^81^ Two years later, the same authors expanded their investigation, continuing to explore the use of date pits in drilling fluids.

In their subsequent study, a modified chemical pretreatment was employed. Date seeds were hydrolyzed in an NaOH solution, followed by agitation, freezing, and thawing. The additive was again incorporated into a bentonite-based fluid containing 350 mL of tap water and 20 g of bentonite. At an optimal concentration of 5 mL, the additive reduced the fluid density from 8.6 to 7.1 ppg, corresponding to a 17.44% reduction.^82^ These studies are noteworthy for introducing a novel, environmentally friendly additive with strong potential for density reduction. However, the additive extraction and preparation methodology presents significant challenges. While successful on a pilot scale, large-scale industrial production may be difficult to implement. Moreover, the fluid formulations tested were relatively simple, consisting only of water and bentonite. In practical drilling operations—where formulations often include more than ten components—the performance and stability of date pit additives should be further evaluated to determine whether comparable density reductions can be achieved under realistic field conditions.

### 5.2 Low-Density Polymers

The use of polymers in drilling fluids has increased over time and has become highly attractive due to their inert nature and generally low density. Recent studies by Hakim et al.^101^ and Yeu et al.^100^ explored the application of polyethylene and polypropylene to improve well cleaning and drill cuttings’ transport efficiency. Although these investigations did not primarily focus on density reduction, noteworthy observations were reported. The polymers exhibited low densities (0.92–0.95 g/cm^3^), and the authors observed that additional bentonite was required to maintain a constant fluid density of 10 ppg as polymer concentration increased. This finding suggests that the polymers effectively contributed to lowering the overall fluid density.^100,101^ These results suggest potential directions for future research on polymer-based additives specifically targeting density reduction in drilling fluids.

Although outside the primary decade framework of this review, the foundational work of Wang et al.^113^ provides essential baseline mechanisms frequently cited in recent literature (2015–2026). In their study, cross-linked polystyrene microspheres (CPS) were developed through a modified soap-free emulsion polymerization method to serve as density-reducing agents. Using a xanthan gum solution to simulate drilling fluid behavior, they reported that CPS reduced the solution density to approximately 6.00 ppg (0.72 g/cm^3^) at 25 °C and 7.33 ppg (0.88 g/cm^3^) at 100 °C with a 20% CPS concentration.^113^ This research demonstrates that polymers can serve as lightweight, chemically inert materials with considerable promise for drilling fluid applications.

Although polymer-based additives exhibit great potential, further investigation is required to validate their performance under realistic drilling conditions. In the case of Wang et al.,^113^ experiments were conducted using a simplified system containing only xanthan gum. However, actual drilling fluids often include ten or more components, and the complex interactions among polymers and these additives may limit the expected density reduction or alter other critical properties. Additionally, scaling up this technology for field use presents challenges related to the mechanical and chemical stability of the polymers as well as degradation under high temperature, pressure, and shear conditions. Despite these challenges, polymeric additives remain a promising avenue for future research and technological development in low-density drilling fluids.

### 5.3 Bio-Enhancing Additives

There is an increasing trend in drilling operations toward the use of biodegradable and environmentally friendly additives, such as *broad bean peel powder, rice husk, guar gum, castor oil, mandarin peel powder, and green olive pit powder*. A representative example is the study by Awl et al.,^102^ who investigated *broad bean peel powder* (BBPP) as an additive in water-based drilling fluids to improve fluid loss and rheological properties. After collecting, treating, and grinding the broad bean peels, the additive was incorporated into a water-based drilling fluid and tested at various concentrations. The results showed a 10–39% reduction in fluid alkalinity and improvements in rheological parameters, particularly plastic viscosity and gel strength. The filter cake thickness decreased from 1.75 mm to 1.0 mm, and fluid loss was reduced from 20.4 to 13.3 mL. Additionally, the fluid density decreased from 8.6 ppg to 7.8 ppg, corresponding to a 9.3% reduction with the addition of only 3 wt.% BBPP.^102^

A similar investigation was conducted by Al-Hameedi et al.,^84^ who evaluated *mandarin peel powder* (MPP) as an innovative bioadditive. Following a comparable preparation process, the peels were collected, treated, and ground before being incorporated into the drilling fluid. The addition of MPP reduced alkalinity by 20–32% and improved rheological parameters such as plastic viscosity, yield point, and gel strength. Moreover, MPP substantially reduced fluid loss by 44–68% at low concentrations (1–4 wt.%), while improving filter cake quality relative to the reference mud—results consistent with those of Awl et al.^102^ A density reduction of 6.98% was also reported, from 8.6 ppg to 8.0 ppg.^84^

However, in both studies, the authors noted that the apparent density reductions may have resulted from the formation of bubbles and foam during agitation, rather than from the intrinsic properties of the biomaterials.^83,84^ In fact, other research has shown that BBPP may increase fluid density under certain conditions.^114^ This observation raises an interesting hypothesis: while these bioadditives may not directly reduce density, they might contain chemical constituents that promote foaming or surfactant-like behavior. Such properties warrant further investigation, both as potential low-density solids and as precursors for surfactant or foam-generating systems—fields that hold promising avenues for future research.

Another noteworthy contribution was presented by Hasan et al.,^103^ who examined *guar gum* and *castor oil* as biolubricants in water-based drilling fluids, focusing on rheology and filtration performance. Castor oil maintained stable rheological properties after dynamic aging at elevated temperatures, whereas guar gum exhibited significant degradation, indicating partial thermal damage. Fluid loss was satisfactory before aging, although results varied after hot rolling. While density was not the main focus, the findings were noteworthy: with 0.1 wt.% castor oil, the fluid density decreased from 10.4 ppg to 9.6 ppg and further to approximately 9.3 ppg after thermal aging—a substantial reduction.^103^ In addition to improving rheological and filtration performance, these oils demonstrated potential as effective density-reducing agents, meriting further investigation into their stability and mechanisms of action.

Further studies by Al-Hameedi et al.^83^ and Mech et al.,^98^ explored bioadditives derived from *green olive pit powder* (GOPP) and *rice husk* (RH), respectively. Both additives improved rheological behavior and filtration control, with GOPP also enhancing yield point and gel strength. However, density variations were negligible, with RH and GOPP formulations showing changes of approximately ± 1%, suggesting limited direct influence on fluid weight negligible.^83,98^

It is important to note that many bioadditives do not intrinsically reduce density. Reported density reductions often result from aeration during mixing or surfactant-like behavior rather than from a low solid density. Thus, their primary contribution may lie in rheological or filtration improvements rather than in direct density reduction. Another critical consideration for these ecological additives is their scalability: large-scale implementation must account for logistical challenges, such as transportation, costs, and practical methods for downhole introduction before field application becomes feasible.

Despite the environmental benefits of bioadditives, several chemical and practical constraints must be considered for their effective application. Because of their organic and biodegradable nature, these materials can be susceptible to chemical degradation through hydrolysis and thermal decomposition under high-temperature drilling conditions. In addition, bioadditives may be prone to microbial activity, particularly in aqueous environments, which can compromise fluid stability.^115,116^ Thus, despite the considerable potential of these green additives, chemical modifications are still required to enhance their stability and applicability to drilling fluids.

### 5.4 Hollow-Glass Microspheres (HGSs)

Hollow glass microspheres (HGSs) are among the most extensively studied and commonly utilized additives for density reduction in drilling fluids, with a significant portion of the literature centered on their formulation and optimization. Lim et al.^106,107^ investigated the optimization of lightweight biopolymer drilling fluids incorporating HGS using Response Surface Methodology (RSM). The authors systematically varied the concentrations of four components—HGS, clay, xanthan gum, and starch—to assess their influence on fluid density, plastic viscosity (PV), and yield point (YP). Through RSM analysis, they identified the optimal formulation and validated their predictive models experimentally. The results were highly satisfactory, with the lowest fluid density recorded at 6.26 ppg, demonstrating the strong effectiveness of HGS in reducing drilling fluid density.^106,107^

A related study by Alawami et al.^108^ further examined the performance of HGS under challenging operational conditions, specifically focusing on their chemical and thermal stability. The research aimed to address the practical difficulties of mud weight reduction in Saudi Arabian fields by assessing the effect of pH and high-pressure, high-temperature (HPHT) aging on HGS-based fluids. Tests were conducted at pH values of 4, 9, and 11, with dynamic aging periods ranging from 1 to 4 days, followed by measurements of rheological properties, pH, and density before and after hot rolling. The results indicated that HGS maintained structural stability across the tested pH range and that the fluids preserved favorable rheological characteristics after thermal exposure. The resulting mud density of 7.6 ppg, though higher than that achieved in Lim et al.,^106^ can be attributed to the study’s focus on evaluating stability rather than minimizing density.^108^

An outstanding review on hollow glass spheres (HGSs) was presented by AlBahrani et al.,^32^ focusing on their reliability as density-reducing agents in drilling fluids and cement slurries. The study evaluated HGS stability across a range of chemical, mechanical, and thermal conditions, investigating their behavior under varying pH values, physical impact forces, and elevated temperatures. The authors reported that HGSs exhibit excellent thermal stability and good chemical resistance under typical downhole pH conditions, with only minor degradation observed in highly alkaline environments (pH > 11). However, mechanical stability emerged as the principal limitation: exposure to impact and shear forces led to microsphere breakage, which in turn increased the overall fluid density.^32^ This mechanical vulnerability remains one of the major challenges associated with the field use of HGSs in drilling operations.

In a related experimental study, AlBahrani et al.^110^ investigated the degradation behavior of drilling fluids containing HGS under simulated downhole conditions. A novel feature of their experimental design was the inclusion of a loose steel cylinder inside the hot-rolling cell to replicate the mechanical impacts that occur during circulation and tool movement. Four fluid formulations containing HGS with different pressure-collapse ratings were subjected to 4 days of dynamic HPHT aging. The lowest density achieved among the formulations was 6.95 ppg. The results revealed significant degradation of HGS due to impact forces, leading to measurable increases in the fluid density. In the most severe case, the density rose by 1.87 ppg, corresponding to 26% of breakage, whereas the most resilient formulation exhibited only a 0.51 ppg increase (approximately 7% of breakage). These findings demonstrate that maintaining HGS integrity is critical for consistent fluid performance and that continuous monitoring and management are essential during field operations.^110^

Similarly, Kirgil et al.^111^ investigated the survival rate of hollow glass spheres (HGSs), focusing on their flow behavior and mechanical durability under varying flow conditions. The experiments were conducted using three types of HGS with different compressive strengths and specific gravities, circulated within a closed-loop flow system. The results indicated that sphere breakage occurred progressively due to cyclic impact fatigue, and the survival rate was strongly influenced by operational parameters such as nozzle spacing, fluid velocity, and HGS concentration. Breakage increased by approximately 10–20% as nozzle spacing decreased, whereas higher HGS concentrations mitigated damage by reducing collision frequency. Although the testing methodology differed, the observed breakage rates were comparable to those reported by AlBahrani et al.,^110^ ranging from 7% to 26%.^110,111^

A notable field-scale application was reported by Askø et al.,^112^ who developed, qualified, and implemented an oil-based low-density drilling fluid containing HGS for drilling a horizontal well in the depleted limestone reservoirs of the Valhall field, Norway. The base formulation was modified by incorporating HGS with a compressive strength of 19,000 psi to achieve target densities between 6.5 and 7.1 ppg. The equivalent circulating density (ECD), which represents the effective fluid density in the wellbore during circulation, increased from 6.9 to 8.0 ppg at total depth because of solids accumulation. The HGS maintained their structural integrity throughout the operation, enabling the successful drilling and completion of a 1750 m horizontal well. The fluid showed stable rheological properties, effective filtration control, and efficient hole cleaning, while sustaining an ROP of approximately 30 m/h. Field observations suggested that rates of 40 to 50 m/h could have been achieved.^112^ These results demonstrate the applicability of HGS-based low-density oil muds in depleted reservoirs, although continuous monitoring of ECD and solids accumulation remains necessary during drilling operations.^112^

Another field-scale investigation employing oil-based drilling fluids was conducted by Minhas et al.,^34^ in three wells within the Montney Formation, Kakwa field, Canada. The study aimed to evaluate hollow glass spheres (HGSs) as a cost-effective alternative to nitrogen for reducing the hydrostatic pressure of inverted emulsion systems in deep, high-pressure formations, where hydrostatic pressures ranged from approximately 2,000 to 3,300 psi. The results demonstrated that the incorporation of HGSs provided an economical and efficient means of mitigating circulation losses. In wells B and C, fluid losses were dramatically reduced relative to those of the reference well (well A), while the ROP increased 36%, contributing to overall cost savings. In well C, the drilling fluid density was successfully reduced to 6.88 ppg, and the HGSs maintained structural integrity throughout the operation, with no significant breakage observed. Despite these positive outcomes, the successful implementation of the technology required careful solid control management to maintain the desired HGS concentration in the active fluid system and ensure consistent density reduction throughout the operation.^34^

A recent and highly innovative study by Chen et al.^33^ developed and evaluated an ultralow-density drilling fluid that leveraged the synergistic effects of foaming agents and HGS for use in formations with low-pressure coefficients. The resulting system achieved a density of 5.84 ppg—the lowest reported among all reviewed studies. Compared to fluids containing only HGS, the combined formulation exhibited enhanced sealing and pressure-bearing capacity, withstanding over 12 MPa in leakage tests and achieving a 21.37% density reduction. The fluid also presented strong thermal and mechanical stability after aging at 80 °C, reduced filtrate loss, and the formation of a mesh-like structure linking the foam matrix with the glass spheres. Moreover, the ultralow-density drilling (ULDD) fluid showed excellent reservoir protection, yielding an average permeability recovery of 85%.^33^ Although the introduction of foam adds operational complexity due to its two-phase, compressible nature, the demonstrated synergy between foamed systems and HGS is highly promising. Extending this concept to oil-based formulations could enable the development of even lower-density systems in the future.

Another noteworthy contribution was presented by Kutlu et al.,^80^ who examined the influence of HGS addition on the density and hydraulic drag characteristics of drilling fluids. Their results revealed a linear decrease in density with increasing HGS concentration, reaching a minimum of 6.3 ppg at 40% HGS loading, along with a high microsphere survival rate of 93% under the manufacturer’s nominal collapse pressure.^80^ These findings further confirm the effectiveness of HGS as a robust additive for density reduction in drilling fluids.

### 5.5 Additive Comparison

Overall, hollow glass spheres (HGSs) represent a well-established density-reduction technology for low-density drilling fluids, with performance demonstrated in both laboratory studies and field applications. Because HGSs behave as incompressible lightweight solids, their addition reduces fluid density while typically increasing plastic viscosity (PV) and yield point (YP) as concentration increases. Reported results also indicate that HGSs can contribute to fluid-loss reduction, with reductions of approximately 25% and up to about 30% in formulations optimized with foaming agents.^33,117^ Polymeric additives have also shown potential for low-density drilling fluid applications. Reported results indicate that polystyrene microspheres can reduce fluid density by approximately 19.13%, while polyethylene spheres can decrease the HPHT fluid loss by about 29% without significantly affecting baseline rheological properties. However, other synthetic polymeric particles, such as polyethylene and polypropylene, may slightly increase plastic viscosity while providing limited filtration-control performance. Although these materials offer low intrinsic density and tunable properties, their application remains mostly restricted to laboratory-scale studies.^100,101^

Date-pit-derived additives have shown promising potential as sustainable materials for low-density drilling-fluid formulations, with reported density reductions of approximately 21%. Depending on the chemical treatment applied, these additives may reduce fluid loss by about 35% through the formation of a low-permeability filter cake. In other cases, however, they may increase filtrate volume by up to 100% while producing a thicker filter cake. This behavior may still be relevant for selected underbalanced drilling applications, where controlled bridging or sealing effects can be beneficial. Nevertheless, challenges related to large-scale production, formulation consistency, and field validation remain.^81,82^ In contrast, most other biobased additives primarily improve rheological and filtration properties, with density-reduction effects that are often secondary or indirect. Organic additives such as MPP have been reported to reduce fluid loss by 8–68%, with the highest reduction associated with improved filter cake quality compared with conventional chemical polymers such as PAC-LV. The rheological effects of these biomaterials vary considerably. For example, broad bean peel can increase plastic viscosity (PV) by up to 75% while sharply reducing yield point (YP), whereas green olive pits can increase YP by up to 172% with minimal effect on PV.^83,84,102^ Table 7 presents a comparative summary of the main additive categories discussed in this section.

**Table 7:** Summary of Additive Types, Density Reduction Range, Main Advantages, and Rheological and Filtration Parameters^a^

**Additive Type**	**Lowest Density (ppg)**	**Density Reduction**	**Main Advantages**	**Rheology**	**Filtration**
Date pits	6.3	17–21%	Renewable, low-cost, sustainable	N.R.	35% reduction
Polymeric additives	6.0	19.13%	Low intrinsic density; tunable properties	Slightly increase/No change	29% reduction
Biobased additives	7.8	6.98–9.30%	Sustainable; improves rheology and filtration	Increase/No change	8–68% reduction
Hollow glass microspheres (HGSs)	6.26	16.71–22.9%	Proven density reduction; thermal stability; field applications available	Increase as concentration increases	25% reduction

a N.R.: Not reported.

To provide a comprehensive overview of the performance of different material classes used for drilling fluid density reduction, Table 8 summarizes their main properties, stability profiles, economic implications, environmental impact, and field applications. By aligning laboratory findings with field-based considerations, the table facilitates a clearer evaluation of trade-offs among cost, performance, and stability. This summary aims to offer a concise and practical reference for field engineers and professionals involved in the selection, formulation, and application of low-density drilling fluids.

**Table 8:** Comparative Assessment of Density-Reduction Technology, Stability, Cost, Operational Trade-Offs, Environmental Impact, and Field Application with Different Additives

**Technology**	**Density (ppg)**	**Stability**	**Cost**	**Trade-offs**	**Eco-Impact**	**Field application**	**Reference**
HGS	6.26–6.95	Good thermal/chemical/mechanical stability	High	Ideal when incompressibility is required; careful handling needed to avoid breakage.	Low (inert material)	Several field applications reported	^32,34,108,112^
HGS + Foam	5.84	Depends on foam quality	Very high	Achieves ultralow densities but adds control and cost challenges.	Moderate (depends on surfactant formulation and gas source)	Limited to specific UBD applications	^33^
Polymeric Microspheres	6.0–7.4	Variable; may degrade thermally	Medium	Promising materials, but HPHT and chemical compatibility need further validation.	Moderate (depends on polymer type)	Emerging technology; limited field validation	^100,101,113^
Bio-Based Additives	6.3–8.0	Low–Medium; depends on pretreatment and purity	Low	Eco-friendly and inexpensive, but variable performance and limited field data.	Low	Mostly laboratory and pilot-scale studies	^118–127^

As summarized in Table 8, hollow glass spheres (HGSs) remain the benchmark additive for formulating low-density, incompressible drilling fluids that exhibit favorable rheological behavior and thermal–chemical stability. However, their relatively high cost and mechanical fragility continue to motivate the search for alternative materials. Polymeric microspheres represent a promising class of density reduction additives, offering tunable properties and lower density targets, although their long-term stability and compatibility with complex mud chemistries require further investigation. Bio-based additives, while attractive due to their low cost and environmental advantages, still yield variable results and have been tested under limited field conditions.

Ultimately, the choice of additive depends on the balance among technical requirements, economic constraints, and environmental priorities for each drilling application. It is important to indicate that research on low-density additives remains in its early stages; the available studies represent only the initial steps toward exploring this niche area of drilling fluid engineering. The field holds considerable potential for discovery and innovation, with numerous additive types yet to be characterized—indicating that continued research is both necessary and promising for future development and industrial applications.

## 6 Trends and Future Perspectives

Further advances in low-density drilling fluids will depend on sustained research efforts and close collaboration between academia and industry. Expanding research on novel materials, hybrid systems, and field-scale validation will support the development of safer, more efficient, and more sustainable drilling operations. These efforts may also foster technological innovation and accelerate the translation of emerging concepts to practical field applications. Future research and development in this field are expected to advance along four main directions:

### 6.1 Development and Discovery of Low-Density Materials

The creation of new lightweight additives, such as glass and polymer microspheres, remains a key research priority. These materials can be engineered to meet specific operational demands, while novel compounds from other disciplines may also be adapted as drilling fluid additives to achieve further density reduction.

### 6.2 Field-Scale Validation and Performance Testing

Greater emphasis must be placed on field-oriented studies that evaluate additive behavior under dynamic downhole conditions. Real-world testing is essential to bridging the current gap between laboratory performance and full-scale operational deployment.

### 6.3 Sustainability and Green Formulation Design

The increasing environmental focus of the oil and gas industry underlines the need for eco-friendly, biodegradable additives. This trend is already reflected in the predominance of water-based drilling fluids and the growing number of studies investigating biobased materials, which combine technical performance with reduced ecological impact.

### 6.4 Transition from Compressible to Incompressible Systems

In applications where ultralow densities are not required, a progressive shift from air, mist, or foam drilling toward incompressible low-density systems is anticipated. This approach minimizes the operational challenges and safety risks associated with gas-based fluids by employing tailored additives that reduce the density without the complexity of two-phase flow systems.

A key consideration for future research is the reevaluation of oil-based drilling fluids (OBFs). While the current literature is predominantly focused on water-based systems, reflecting the industry trend toward lower toxicity and improved environmental performance, oil-based fluids inherently offer advantages for density reduction due to the lower specific gravity of oil compared to water. The development of environmentally friendly base oils with reduced toxicity may, therefore, renew interest in OBFs for drilling applications. In this context, the integration of next-generation oil-based systems with advanced density-reducing additives, such as hollow glass spheres (HGSs) or polymeric microspheres, could enable ultralow-density fluid formulations and represent a significant step forward in lightweight drilling fluid technology. Future developments are, therefore, expected to focus on oil-based systems that achieve an improved balance among performance, safety, and environmental compliance.

Another important research direction involves the synergistic combination of low-density technologies. Previous studies have demonstrated the feasibility of hybrid systems, such as HGS-based foamed fluids, which combine the stability of incompressible phases with the density reduction provided by gas incorporation. This approach warrants further investigation to optimize formulation design for enhanced density reduction and operational stability. Although multiphase systems introduce additional formulation complexity, their potential benefits—including improved safety margins and reduced fire and explosion risks compared to gas-only systems—make them attractive for future applications. Ultimately, the selection of hybrid strategies should be guided by operational constraints and a clear understanding of the trade-offs among density reduction, stability, and environmental impact.

Nanotechnology also represents a promising approach for enhancing the drilling fluid performance. Although nanoparticles do not directly reduce the fluid density, their high surface area enables performance improvements at low concentrations, thereby minimizing undesirable density increases. Their main benefits include improved rheological control, enhanced thermal stability under HPHT conditions, and the formation of thin, low-permeability filter cakes that improve the filtration efficiency. Furthermore, the synergistic use of nanoparticles with biodegradable materials, such as plant-derived waste powders, may further enhance fluid performance while reducing environmental impact.^128–130^

A key research direction is the development of multifunctional additives capable of simultaneously reducing drilling fluid density and providing additional operational functions, including shale inhibition, fluid loss control, bit lubrication, thermal stability, and wellbore strengthening. Such multipurpose additives may simplify fluid formulations, reduce chemical consumption, and improve overall operational efficiency by decreasing the number of required components. These additives are expected to play an increasingly important role in drilling operations, particularly in HPHT environments, where conventional additives may undergo degradation, loss of integrity, and changes in rheological performance.^131^

The increasing digitalization of drilling operations, driven by artificial intelligence, big data, cloud computing, and high-speed communication networks (4G/5G), opens new opportunities for the development of intelligent drilling fluids. Future formulations may incorporate smart additives and responsive materials capable of self-monitoring, self-adjustment, and adaptation to dynamic downhole conditions in coordination with real-time monitoring systems. Although these technologies remain at an early conceptual stage, their integration with advanced decision-support systems may significantly improve operational safety and efficiency by enabling early detection and mitigation of downhole complications.^132,133^

## 7 Conclusions

This review critically examines density-reducing additives and low-density drilling-fluid technologies, with attention to their mechanisms, operational applicability, limitations, and remaining development needs. From 54 articles initially retrieved from ScienceDirect, Web of Science, and Scopus, 28 publications were retained within the defined scope, including 23 experimental studies and 5 review papers. The reviewed literature showed a strong predominance of water-based systems, which represented approximately 91% of the reported formulations, whereas oil-based systems accounted for only 9%; this imbalance indicates an important research gap, given the intrinsically lower density of oil-based fluids. Among the investigated materials, hollow glass spheres (HGSs) remain the most established approach for low-density, incompressible drilling fluids, with reported final densities of approximately 6.26–6.95 ppg and density reductions ranging from 16.71% to 22.9%. The lowest density identified in the reviewed studies was 5.84 ppg, obtained using a hybrid formulation combining HGS and foaming agents, indicating the feasibility of synergistic approaches for ultralow-density fluid design. Polymeric microspheres and biobased additives have also shown technical potential, with date-pit-derived materials achieving density reductions of approximately 17–21%; however, most other bio-based additives appear to act mainly as rheology and filtration modifiers, with density-reduction effects that are often modest or indirect. Taken together, these results suggest that research on density-reducing additives remains limited but technically relevant and that future work should prioritize field-scale validation, compatibility with realistic drilling-fluid formulations, long-term stability under HPHT and high-shear conditions, and the development of hybrid, multifunctional, and environmentally responsible lightweight drilling fluids.
